# Does Sleep Quality Affect IVF Outcomes?

**DOI:** 10.5935/1518-0557.20250048

**Published:** 2025

**Authors:** Lara S. Pamfilio, Elaine C. F. de Oliveira, Frederico T. R. Sousa, Maira Casalechi, Fernando M. Reis

**Affiliations:** 1 Department of Obstetrics and Gynecology, Universidade Federal de Minas Gerais, Belo Horizonte, Brazil; 2 Fondazione IRCCS Ca’Granda Ospedale Maggiore Policlinico, Milan, Italy

**Keywords:** sleep, IVF outcomes, pregnancy rates embryo implantation rates

## Abstract

**Objective:** This narrative review aims to summarize the current understanding of the relationship between sleep quality and outcomes in in vitro fertilization (IVF) treatments. An intricate connection links sleep and reproductive health. Sleep affects hormonal regulation and reproductive processes by several physiological mechanisms, underscoring the importance of considering sleep as a modifiable factor in fertility treatments. Evidence suggests that sleep habits and quality can affect IVF outcomes like clinical pregnancy and live birth rates. However, the heterogeneity in study designs and methodologies poses challenges in drawing definitive conclusions. Further research should employ standardized and objective measures of sleep quality to deepen our understanding and improve clinical guidance for couples undergoing IVF. The potential for behavioral interventions to enhance sleep quality offers a promising opportunity for improving fertility outcomes, warranting more focused investigations in this area.

## INTRODUCTION

Infertility is a growing health problem in recent times, which affects approximately 15% of the adult population worldwide (Mascarenhas *et al*., 2012) and represents a substantial burden to health care systems (Oliveira *et al*., 2021). Among several factors that stand out for this scenario, advanced female age is one of the main ones. Furthermore, lifestyle factors like unbalanced diet, smoking, excessive alcohol consumption, sedentary habits and poor sleep quality also play an important role in this context (Rotimi & Singh, 2024).

Inadequate sleep has been linked to numerous diseases and chronic conditions, including diabetes, obesity and depression, which can directly or indirectly impact fertility (Caetano *et al*., 2021). Circadian cycles interfere with the homeostasis of multiple systems, as the sleep-wake cycle modulates essential processes such body temperature control, autonomic functions, and hormonal secretion (Sciarra *et al*., 2020).

Sleep is a cyclical physiological state, structured in 4 to 6 cycles lasting 90 to 100 minutes each. In human, these cycles are divided into 5 fundamental stages: 4 stages of non-REM sleep (I to IV) and REM sleep (Berry *et al*., 2020). The sleep-wake cycle can be disturbed by various incidents like pain, stress, anxiety, and also by lifestyle factors like a diet with a high glycemic index, nighttime exercising, the consumption of alcohol or stimulants and the abuse of electronic devices (Sejbuk *et al*., 2022).

The “Clock” genes and their molecular products control circadian rhythms and have great importance for the regulation of female reproductive processes. These genes regulate gonadotropin-releasing hormone (GnRH) secretion via two neuronal pathways: directly, through vasoactive intestinal peptide (VIP), and indirectly, through arginine-vasopressin (AVP). Furthermore, they play a role in the expression of estrogen receptors in target tissues and indirectly affect the periodic differentiation of the endometrium that makes it receptive to embryo implantation and early pregnancy (Beroukhim *et al*., 2022). Sleep cycles have a great influence on women’s hormonal secretion, which, in turn, has an essential role in maintaining the proper functioning of several organic systems, including the reproductive system (Caetano *et al*., 2021).

In view of the importance of sleep for natural fertility, it has been hypothesized that sleep quality might also affect the ovarian and uterine responses to fertility treatments like in vitro fertilization (IVF). Therefore, this review intends to evaluate the available evidence about the impact of sleep quality on IVF outcomes.

## METHODS

A comprehensive and non-systematic search was conducted to investigate the available evidence on the association between sleep quality, embryo implantation rates, and pregnancy outcomes in women undergoing IVF. The search strategy included the following keywords: (“in vitro fertilization” OR “IVF”) AND (“sleep quality” OR “sleep disturbances” OR “poor sleep”) AND (“embryo implantation” OR “implantation success” OR “embryo transfer outcome”) AND (“pregnancy rates” OR “pregnancy outcomes”). The online databases PubMed, Embase, Cochrane, and SCIELO were consulted, with searches conducted up to October 2024. Only articles published in English were included. Additionally, a manual review of the references cited in the included articles was performed to identify other relevant studies.

The following types of publications were excluded: letters to the editor, comments, literature reviews without quantitative data, case series, and cross-sectional studies.

Two reviewers (LSP and ECFO) independently screened the titles and abstracts of articles retrieved through the predefined search strategy and applied the exclusion criteria. Full texts of potentially eligible studies were then obtained and independently reviewed to assess eligibility based on the same criteria. Any disagreements between the reviewers were resolved through discussion and consensus.

## RESULTS

### Influence of sleep on women’s hormonal axes

**Figure 1:**
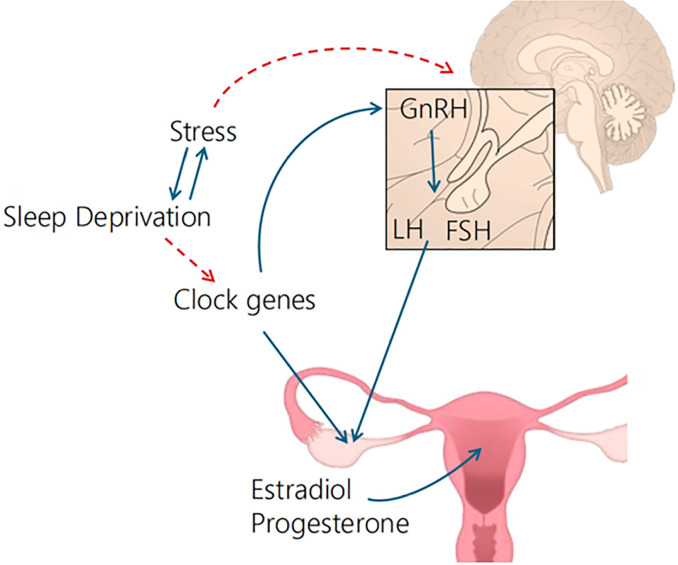
Negative impact of sleep deprivation on the female gonadotropic axis and reproductive function.

Sleep, especially during the slow-wave phase, significantly influences gonadotropin release. The Clock genes modulate the pulsatility of GnRH and LH, in addition to directly affecting steroidogenesis and ovarian reserve (Figure 1). Among the Clock genes, *Bmal1* regulates the synthesis of estradiol and progesterone, which in turn regulate the expression of other sleep-related genes, such as *Per1* and *Per2* (Sciarra *et al*., 2020). Therefore, disturbances in the sleep cycle cause dysregulation on the hypothalamic-pituitary-gonadal axis, which contributes to reproductive dysfunctions.

A prospective study analyzing daily urinary samples from women with regular menstrual cycles subdivided according to their sleep patterns showed slightly higher FSH levels in long-time sleepers than in short-time sleepers (Touzet *et al*., 2002). However, findings regarding estradiol levels are controversial. Data from 213 women of the cross-sectional Study of Health in Pomerania showed a positive association of estradiol levels with diurnal sleepiness, an indicator of poor sleep quality (Kische *et al*., 2016). In contrast, a prospective cohort of 259 healthy, regularly menstruating women noted lower estradiol levels in short sleepers (<7 hours per night on average) (Michels *et al*., 2020). As for progesterone, a reciprocal influence is observed: reduced hours of sleep imply lower progesterone levels both directly and because of the resulting stress. On the other hand, progesterone is a sleep inducer and has anxiolytic properties (Beroukhim *et al*., 2022).

Other hormones like thyroxin (T4) and thyrotropin (TSH) play an important role in menstrual regularity, ovulation and pregnancy maintenance. Evidence suggests that acute sleep deprivation may increase TSH levels (Kuhs *et al*., 1996) whereas chronic sleep deprivation may cause a mild TSH reduction accompanied by a decrease of free T4 levels (Kessler *et al*., 2010). As for prolactin and cortisol, the scarce evidence in women suggests that partial sleep deprivation does not affect their serum levels (Kuhs *et al*., 1996), although both hormones increase in situations of stress that can arise from sleep disturbances (Beroukhim *et al.*, 2022). Melatonin, a hormone secreted by the pineal gland, plays an important role synchronizing the circadian rhythms and has antioxidant, anti-inflammatory and anti-apoptotic effects. Short-term sleep deprivation reduces melatonin levels (Chen *et al*., 2023), which theoretically may compromise the ovarian follicular environment by increasing oocyte susceptibility to oxidative stress (Caetano *et al*., 2021; Tamura *et al*., 2008).

Studies have shown an association between short sleep duration and menstrual irregularity (Caetano *et al*., 2021; Nam *et al*., 2017). A systematic review found that short periods of sleep (<5 to 6h) can interfere with the menstrual cycle, impair seminal parameters and reduce natural fertility (Caetano *et al*., 2021). Furthermore, poor-quality sleep has been associated with reduced ovarian reserve, menstrual irregularity and infertility (Philipsen *et al*., 2022). This effect seems to be bidirectional, as infertility itself and related treatments can adversely affect sleep. For example, women receiving long protocol of ovarian stimulation have reported more sleep disturbances and higher rates of depression, probably due to greater pituitary suppression, compared to short protocols (Philipsen *et al*., 2022).

### Sleep changes associated with infertility and IVF

Infertility by itself and the pursuit of IVF treatment are significant sources of chronic stress (Sousa *et al*., 2023), which has somatic manifestations like endocrine and autonomic responses along with sleep disturbances (Tsigos *et al*., 2000). A cross-sectional study using the Pittsburgh Sleep Quality Index (PSQI), a self-administered tool for assessment of sleep latency, duration, efficiency and subjective quality, suggested a concerning prevalence of short sleep (56%) and of low sleep efficiency (44%) in women starting an IVF cycle. However, these numbers are difficult to interpret due to the lack of a control group (Huang *et al*., 2019). A similar study in a different population further indicated that sleep quality worsens as the IVF treatment advances (Yanik & Tokat, 2024).

A cross-sectional study involving 595 infertile women undergoing IVF found that poor sleep quality, as assessed by the PSQI, correlated with higher psychological distress scores. This finding suggests that sleep disturbances could exacerbate stress levels, potentially influencing the fertility treatment process (Jiang *et al*., 2024).

Another cross-sectional study investigated the association between sleep quality and ovarian reserve in reproductive-age women. A total of 1,070 participants aged 20-40 years were assessed using the PSQI and biomarkers of ovarian reserve, including antral follicle count (AFC), anti-Müllerian hormone (AMH), and follicle-stimulating hormone (FSH). Results showed that women with poor sleep had significantly lower AMH and AFC levels and higher FSH levels compared to those with better sleep. Multivariate analysis revealed that poor sleepers had a 4.43 times higher likelihood of diminished ovarian reserve than good sleepers, and subgroup analyses indicated that this association was stronger in women under 35 years and those with lower BMI (Lin *et al.*, 2025).

### Relationship between sleep quality and IVF results

A prospective cohort study including 7,847 women seeking IVF treatment showed that good sleep quality was associated with better clinical pregnancy and live birth rates. Specifically, women with good sleep quality achieved a clinical pregnancy rate of 69.3% compared to 65.1% for those with poor sleep quality, and a live birth rate of 50.5% *versus* 45.7%. After adjusting for confounding factors, women with a good sleep quality had a significantly higher probability of achieving a clinical pregnancy and a live birth (Liu *et al*., 2023) (Table 1).

**Table 1:** Summary of studies evaluating the association of sleep quality and IVF outcomes.

Reference/Country	Design	Participants	Instrument	Main Results
Bariya *et al*., 2024 China	Cohort, prospective	Women undergoing IVF (n=174)	PSQI	The number of oocytes retrieved was reduced by 22% in individuals with poor sleep quality. However, none of the PSQI parameters was independently associated with pregnancy.
Liu *et al*., 2023 China	Cohort, prospective	Women undergoing first IVF cycle (n=3183)	PSQI	Live birth rate was higher in the group reporting good sleep (50.5%) than in the group reporting poor sleep (45.7). The difference was still significant after adjusting for potential confounders (RR = 1.12, 95% CI 1.02-1.23).
Philipsen *et al*., 2022 Denmark	Secondary analysis of RCT	Women (n=163) and partners (n=132) undergoing IVF	PSQI	Pregnancy was not significantly associated with sleep quality or duration.
Pimolsri *et al*., 2021 USA	Cohort, prospective	Women undergoing IVF (n=48)	Actigraphy	Cycle cancelation was associated with worse sleep parameters like short duration, nocturnal awakenings and low efficiency. Later sleep midpoint and bedtime were associated with increased odds of cycle cancelation.
Reschini *et al*., 2022 Italy	Cohort, prospective	Women undergoing IVF (n=263)	PSQI	Women who achieved a clinical pregnancy had lower PSQI scores than non-pregnant counterparts. The adjusted OR associated with poor sleep quality (PSQI>5) for clinical pregnancy was 0.50 (95% CI 0.26-0.94).
Walter *et al*., 2022 USA	Cohort, prospective	Women with primary infertility undergoing IVF (n=30)	Home-based night of sleep monitoring using a wireless two sensor system	Live birth rate was 58% in the group with normal breathing and 38% in the group with sleep disordered breathing (*p*=0.24). Intermediate IVF outcomes like number of mature oocytes or embryos transferred were similar in both groups.
Yao *et al*., 2022 China	Cohort, prospective	Women undergoing IVF (n=1276)	PSQI, simplified	Lower number of oocytes (total and mature) associated with short sleep; Lower fertilization rate associated with poor sleep quality; in multivariable analysis, only sleep duration 9 to <10 hours associated with lower odds of pregnancy (OR 0.65, 95% CI 0.44-0.98).

PSQI: Pittsburgh Sleep Quality Index.

Walter *et al*. (2002) explored the effects of sleep-disordered breathing (SDB) on pregnancy outcomes in 30 women undergoing IVF at an academic infertility center. Pregnancy rates were 35% in women with SDB *versus* 58% in women without SDB, but the difference was not significant due to the small sample size (Walter *et al*., 2022). In contrast, a study by Reschini *et al*. (2002) found a significant relationship between sleep quality and IVF success. The study revealed that women with poor sleep quality (PSQI > 5) had lower odds of becoming pregnant, with an adjusted odds ratio of 0.50 (Reschini *et al*., 2022) (Table 1).

Yao *et al*. (2022) found that women sleeping 9 to <10 hours per night experienced decreased chances of clinical pregnancy compared to those sleeping 7 to <8 hours; however, no difference was observed when comparing the first group with the group sleeping ≥ 10 hours per night. The same study found that short sleep duration was associated with fewer oocytes retrieved (total and mature) and with lower fertilization rate (Yao *et al*., 2022) (Table 1). Philipsen *et al.* (2022) investigated sleep quality among 163 women and their partners undergoing assisted reproductive technology treatments (conventional IVF or intracytoplasmic sperm injection - ICSI). The study reported that 91% of the participants had poor sleep quality (PSQI > 5), yet no statistically significant differences in pregnancy outcomes were found between good and poor sleepers. This suggests variability in how sleep quality affects IVF success (Philipsen *et al*., 2022).

Meanwhile, Pimolsri *et al*. (2021) demonstrated that later sleep midpoints and bedtimes significantly increased the odds of not completing IVF cycles, regardless of other factors. More recently, a prospective cohort study including 174 women who completed the PSQI before starting IVF/ICSI observed a 22% reduction of total and mature oocytes retrieved in individuals with subjective sleep quality reported as fairly bad or very bad, after adjusting for potential confounders like age, BMI and parity (Bariya *et al.,* 2024). However, none of the PSQI parameters was independently associated with implantation rate or clinical pregnancy rate (Table 1).

As shown in Table 2, among the studies reporting the absolute number of clinical pregnancies in women with good (PSQI ≤ 5) vs. poor (PSQI >5) sleep quality, the clinical pregnancy rates were 4% to 22% lower in the second group.

**Table 2:** Results of studies that correlated poor sleep, defined as Pittsburgh Sleep Quality Index (PSQI) >5, with clinical pregnancy rates in women undergoing IVF.

Study	Good Sleep		Poor Sleep		Pregnancy Rate Reduction (95% CI)
	n	Clinical Pregnancy Rate	n	Clinical Pregnancy Rate	
Liu *et al*., 2023	2284	69.3%	899	65.1%	4% (1 to 8%)
Philipsen *et al*., 2022	11	72.7%	142	50.7%	22% (-8 to 41%)
Reschini *et al*., 2022	183	35.5%	80	20.0%	15% (4 to 26%)

CI: confidence interval.

## DISCUSSION

The association between sleep quality and infertility, as discussed in various studies, reveals a nuanced relationship that underscores the complex interplay between physiological factors influencing reproductive success. Across multiple studies, poor sleep quality, commonly assessed using the PSQI has been frequently linked to lower pregnancy and live birth rates in women undergoing IVF treatments.

Nonetheless, the current body of evidence on the relationship between sleep and infertility is constrained by notable limitations. Primarily, many studies depend heavily on self-reported sleep characteristics, introducing potential recall bias and inaccuracies due to the subjective nature of participants’ responses. This reliance may not fully capture the complexity and full scope of sleep patterns and their impact on reproductive outcomes. Furthermore, the heterogeneity in study methodologies, sample sizes, and outcome measures significantly hampers the comparability of findings. Additionally, the lack of standardized definitions for sleep quality and the diverse tools employed to assess it further complicate efforts to harmonize results across different studies. Such variability often results in inconsistent outcomes and poses challenges to draw clear and generalizable conclusions regarding the link between sleep and the success of fertility treatments like IVF.

Therefore, while existing studies offer valuable insights, future research should focus on incorporating more objective and quantifiable measures of sleep that could complement self-reported data. Moreover, striving for greater uniformity in study designs and methodologies will enhance the reliability and validity of findings, ultimately leading to a deeper understanding of how sleep quality influences infertility treatments and reproductive outcomes.

## SUMMARY AND CONCLUSIONS

There are many efforts to try to optimize natural fertility and improve the outcomes of IVF treatments. The findings of the studies reviewed here collectively suggest that sleep quality may be associated with fertility outcomes. However, the heterogeneity in study methodologies and outcomes points to the need of further research, employing more rigorous and standardized approaches, to better understand the causal pathways linking sleep quality to reproductive success and for developing targeted interventions to improve sleep in women undergoing fertility treatments.

Since high-quality sleep is essential for overall health and well-being, providing guidance to improve sleep habits is an important aspect of health care. Women pursuing fertility treatments are usually motivated and compliant with health care recommendations. If more robust evidence shows that sleep hygiene and other behavioral interventions to improve sleep quality can positively impact IVF outcomes, these interventions would be relatively easy to implement. Such strategies are safe, cost-effective, and likely to align well with the motivations and goals of patients undergoing fertility treatments.
